# Quantitative proteomic analysis of Rett iPSC-derived neuronal progenitors

**DOI:** 10.1186/s13229-020-00344-3

**Published:** 2020-05-27

**Authors:** Suzy Varderidou-Minasian, Lisa Hinz, Dominique Hagemans, Danielle Posthuma, Maarten Altelaar, Vivi M. Heine

**Affiliations:** 1 Biomolecular Mass Spectrometry and Proteomics, Bijvoet Center for Biomolecular Research and Utrecht Institute for Pharmaceutical Sciences, University of Utrecht, Padualaan 8, 3584 CH Utrecht, The Netherlands; 2 Netherlands Proteomics Center, Padualaan 8, 3584 CH Utrecht, The Netherlands; 3 Department of Complex Trait Genetics, Center for Neurogenomics and Cognitive Research, Amsterdam Neuroscience, Vrije Universiteit Amsterdam, Amsterdam, The Netherlands; 4 Child and Youth Psychiatry, Emma Children’s Hospital, Amsterdam UMC, Amsterdam Neuroscience, Vrije Universiteit Amsterdam, Amsterdam, The Netherlands

**Keywords:** Rett syndrome, iPSC, Neuron differentiation, Quantitative mass spectrometry, TMT-10plex

## Abstract

**Background:**

Rett syndrome (RTT) is a progressive neurodevelopmental disease that is characterized by abnormalities in cognitive, social, and motor skills. RTT is often caused by mutations in the X-linked gene encoding methyl-CpG binding protein 2 (MeCP2). The mechanism by which impaired MeCP2 induces the pathological abnormalities in the brain is not understood. Both patients and mouse models have shown abnormalities at molecular and cellular level before typical RTT-associated symptoms appear. This implies that underlying mechanisms are already affected during neurodevelopmental stages.

**Methods:**

To understand the molecular mechanisms involved in disease onset, we used an RTT patient induced pluripotent stem cell (iPSC)-based model with isogenic controls and performed time-series of proteomic analysis using in-depth high-resolution quantitative mass spectrometry during early stages of neuronal development.

**Results:**

We provide mass spectrometry-based quantitative proteomic data, depth of about 7000 proteins, at neuronal progenitor developmental stages of RTT patient cells and isogenic controls. Our data gives evidence of proteomic alteration at early neurodevelopmental stages, suggesting alterations long before the phase that symptoms of RTT syndrome become apparent. Significant changes are associated with the GO enrichment analysis in biological processes *cell*-*cell adhesion*, *actin cytoskeleton organization*, *neuronal stem cell population maintenance*, and *pituitary gland development*, next to protein changes previously associated with RTT, i.e., dendrite morphology and synaptic deficits. Differential expression increased from early to late neural stem cell phases, although proteins involved in immunity, metabolic processes, and calcium signaling were affected throughout all stages analyzed.

**Limitations:**

The limitation of our study is the number of RTT patients analyzed. As the aim of our study was to investigate a large number of proteins, only one patient was considered, of which 3 different RTT iPSC clones and 3 isogenic control iPSC clones were included. Even though this approach allowed the study of mutation-induced alterations due to the usage of isogenic controls, results should be validated on different RTT patients to suggest common disease mechanisms.

**Conclusions:**

During early neuronal differentiation, there are consistent and time-point specific proteomic alterations in RTT patient cells carrying exons 3–4 deletion in *MECP2*. We found changes in proteins involved in pathway associated with RTT phenotypes, including dendrite morphology and synaptogenesis. Our results provide a valuable resource of proteins and pathways for follow-up studies, investigating common mechanisms involved during early disease stages of RTT syndrome.

## Background

Rett syndrome (RTT) is a severe neurodevelopmental disorder that mainly affects females with a frequency of ~ 1:10,000 [1]. Clinical features of RTT start to present around 6–18 months of age and include deceleration of head growth, abnormalities in cognitive, social and motor skill development, and seizures [2, 3]. Postmortem studies showed increased density of neurons in combination with reduced soma sizes in RTT patient compared to healthy control brains [4, 5]. RTT neurons show a decrease in dendritic branching and a reduced number of dendritic spines and synapses [6, 7]. While studies suggest affected neurodevelopment starting at early stages, the molecular mechanisms underlying neuropathology in RTT is not understood.

In 90–95% of the RTT cases, the disease is caused by dominant loss-of-function mutations in the X-linked gene encoding methyl-CpG binding protein 2 (MeCP2) [8]. Random X chromosome inactivation in females results in somatic mosaics with normal and mutant *MECP2* [9]. Males carrying a *MECP2* mutation are not viable or suffer from severe symptoms and die early in life [10]. MeCP2 is described as a nuclear protein modulating gene expression, via binding to methylated DNA and hundreds of target genes. These modulations take place through direct repression or activation of genes or by means of DNA modulation and secondary gene regulation. Consequently, mutations in *MECP2* lead to miss-regulation of hundreds of genes, including those influencing brain development and neuronal maturation [11–14]. So far, research in RTT focused on genomic and transcriptomic studies [15–17] and less so on proteome changes [18, 19]; although as molecular effectors of cellular processes, these are better predictors of pathological states. Recent advances in mass spectrometry-based proteomics now facilitate the study of global protein expression and quantification [20]. Considering the broad and complex regulating functions of MeCP2, modulating multiple cellular processes, we need insight into the final molecular effectors reflected by perturbation at the protein level to understand pathological states.

**Fig. 1:**
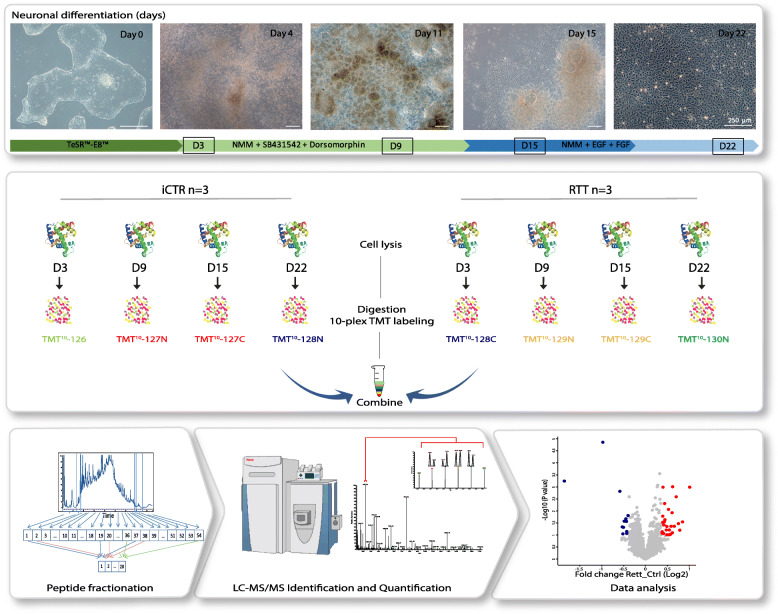
Overview of experimental workflow. iPSC differentiation towards neuronal stem cells. Different colors in arrows indicate change of medium. Squares mark days of sample collection. Samples at indicated time points (four time points) for (three clones) iCTR and (three clones) RTT were processed for proteomic analysis. In total, 24 samples were subjected for tryptic digestion, TMT-based isotope labeling, high-pH fractionation, and LC MS/MS analysis. Different bioinformatic approaches were then used to analyze the data

Here, we used an iPSC-based RTT model and performed proteome analysis on iPSC-derived neuronal stem cells (NES cells) carrying an *MECP2* exons 3–4 mutation [21]. Earlier studies proved that iPSCs from RTT patients reflect disease-specific characteristics, including changes in neuronal differentiation at early stages of development [22, 23]. However, we lack knowledge on the precise molecular mechanisms at the onset of disease. To study early alterations in the proteome of RTT cells compared to isogenic controls (iCTR), we performed a high-resolution mass spectrometry-based quantitative proteomics at different time points during neuronal stem cell development (Fig. 1). We show that the difference between RTT and iCTR, in terms of the number of differentially expressed proteins, begins at early stages and increases at later progenitor stages. Interestingly, a large group of these proteins are involved in cellular processes, implicated in classical features of typical RTT phenotypes, such as dendrite formation and axonal growth. Proteins involved in immunity and metabolic processes are consistently changed between RTT and iCTR at all time points studied. Here, we provide a resource of target proteins and pathways for further studies into molecular mechanisms involved in early RTT disease stages.

## Methods

### Cell culture and isogenic controls

RTT patient fibroblasts were derived from the *Cell lines and DNA bank of Rett syndrome*, *X*-*linked mental retardation*, *and other genetic diseases* at the University Siena in Italy via the Network of Genetic Biobanks Telethon. We used fibroblast lines carrying *MECP2* mutation, showing a deletion in exons 3 and 4 of the *MECP2* gene (RTT Ex3-4), (RTT#2282C2). Fibroblasts were derived frozen, thawed, and expanded in fibroblast medium (DMEM-F12, 20% FBS, 1% NEAA, 1% Pen/Strep, 50 μM β-Mercaptoethanol). To generate pure RTT, i.e., cells expressing affected X chromosome, and isogenic control, i.e., cells expressing the healthy X chromosome, fibroblasts were detached from cell culture plate, and single fibroblasts were seeded in a 96-well plate. Cells were further expanded and characterized for their MeCP2 state by immunocytochemistry and PCR [24]. All of our experiments were exempt from the approval of the institutional review board.

### Reprogramming

Reprogramming of fibroblasts was performed as described before [24]. In brief, fibroblasts were detached from cell culture plate and washed with PBS. 4 × 10^5^ cells were resuspended in 400 μl Gene Pulser® Electroporation Buffer Reagent (BioRad) with 23.4 μg of each episomal plasmid (Addgene, Plasdmid #27078, #27080, #27076), containing the reprogramming genes *OCT4*, *SOX2*, *KLF4*, and *C-MYC*. Cell solution was carefully mixed and electroporated with three pulses of 1.6 kV, capacitance of 3 μF and a resistance of 400 Ω (Gene Pulser II (BioRad)). Fibroblasts were left for recovery in Fibroblast medium without antibiotics, containing 10 μM Rock inhibitor (Y-27632). After cells reached a confluence of 60–70%, medium was changed to TeSR™-E7™ (STEMCELL). Colonies appeared after 21–28 days. These were picked manually and maintained in TeSR™-E8™ (STEMCELL). iPSC lines were characterized for pluripotency [24]. Six iPSC lines derived from one individual were selected and used in the present study, three iCTR clones and three RTT Ex3-4 clones.

### Differentiation of neuronal stem cells

The 6 iPSC lines were differentiated towards neuronal stem cells as described before [21, 25]. As described in the paper by Shi et al., this protocol of cortical neurogenesis follows the same temporal order as occurs in vivo. iPSCs were plated in high density on Geltrex®-coated wells of a 12-well plate in TeSR™-E8™ with 10 μM Rock inhibitor. Medium was changed daily for 2 days. Afterwards, half of the medium was changed daily with Neuro-Maintenance-Medium (NMM) (1:1 DMEM/F12+GlutaMAX:Neurobasal Medium, 1x B27, 1xN2, 2.5 μg/ml insulin, 1.5 mM L-glutamin, 100 μM NEAA, 50 μM 2-mercaptoethanol, 1% penicillin/streptomycin) containing 1 μM dorsomorphin and 10 μM SB431542 up to day 12. At days 10–12, rosette structures appeared, which were manually picked and further cultured on Poly-L-Ornithin (0.01%)/laminin (20 μg/ml) coated cell culture plates in NMM medium containing EGF (20 ng/ml) and FGF-2 (20 ng/ml). Half of medium was changed daily and cells were cultured up to day 22.

### Immunocytochemistry

To perform immunocytochemistry, cells were fixated with 4% paraformaldehyde and blocked with blocking buffer containing 5% normal goat serum (Gibco®), 0.1% bovine serum albumin (SigmaAldrich) and 0.3% Triton X-100 (SigmaAldrich). Primary antibody incubation for MeCP2 (D4F3, CellSignaling, 1:200, rabbit), OCT3/4 (C-10, Santa Cruz, 1:1000, mouse), SSEA4 (Developmental Studies Hybridoma Bank, 1:50, mouse), TRA1-60 (Santa Cruz, 1:200, mouse), TRA1-81 (Millipore, 1:250, mouse) and SOX2 (Millipore, 1:1000, rabbit) was performed in blocking buffer over night at 4 °C. Next day, cells were washed, and secondary antibody Alexa Fluor® 488 (ThermoFisher, 1:1000, mouse or rabbit) and Alexa Fluor® 594 (ThermoFisher, 1:1000, mouse or rabbit) were applied in blocking buffer for 1 h at room temperature. To identify cell nuclei, DAPI was used for 5 min before cells were mounted with Fluoromount™ (Sigma-Aldrich).

### RNA collection, sequencing, and PCR analysis

To isolate RNA samples, standard TRIzol®-Chloroform isolation was done. RNA was stored at – 80 °C until further processing. For PCR analysis, RT-PCR was performed. cDNA was synthesized by using SuperScriptIV-Kit (ThermoFisher) following manufacturer’s recommendations and could be stored until further processing at – 20 °C. To perform PCR, different primer sets were used (Table 1) and PCR was executed with Phire Hot Start II DNA Polymerase (ThermoFisher).

**Table 1:** Primers used for iPSC characterization

*OCT3/4*	Fwd:	GAC AGG GGG AGG GGA GGA GCT AGG
	Rev:	CTT CCC TCC AAC CAG TTG CCC CAA AC
*SOX2*	Fwd:	GGG AAA TGG GAG GGG TGC AAA AGA GG
	Rev:	TTG CGT GAG TGT GGA TGG GAT TGG TG
*NANOG*	Fwd:	CAG CCC CGA TTC TTC CAC CAG TCC C
	Rev:	CGG AAG ATT CCC AGT CGG GTT CAC C
*C-MYC*	Fwd:	GCG TCC TGG GAA GGG AGA TCC GGA GC
	Rev:	TTG AGG GGC ATC GTC GCG GGA GGC TG
*TDGF1*	Fwd:	TGC TGC TCA CAG GGC CCG ATA CTT C
	Rev:	TCC TTT CGA GCT CAG TGC ACC ACA AAA C
*UTF1*	Fwd:	CAG ATC CTA AAC AGC TCG CAG AAT
	Rev:	GCG TAC GCA AAT TAA AGT CCA GA
*DNMT3B*	Fwd:	CAG GAG ACC TAC CCT CCA CA
	Rev:	TGT CTG AAT TCC CGT TCT CC
*MECP2 (Set 1)*	Fwd:	GGA GAA AAG TCC TGG AAG C
	Rev:	CTT CAC GGC TTT CTT TTT GG
*MECP2 (Set 2)*	Fwd:	CACGGAAGCTTAAGCAAAGG
	Rev:	CTGGAGCTTTGGGAGATTTG
*EIF4G2*	Rev:	CTT CAC GGC TTT CTT TTT GG
	Rev:	AGT TGT TTG CTG CGG AGT TGT CAT CTC GTC

### Western blotting

Frozen cell pellets were lysed by adding WB-Lysate buffer (50 mM Hepes ph 7.5, 150 mM NaCl, 1 mM EDTA, 2.5 mM EGTA, 0.1% TritonX-100, 10% glycerol, 1 mM DTT). To determine protein concentration, Bradford-Test was performed, and 30 μg of sample was used. For SDS-PAGE, pre-casted gels were used (Biorad) and ran in 10× Tris/glycine buffer for Western Blots and Native Gels (Biorad #1610734). Gels were blotted in tank-blotter (Biorad) on PVDF membranes (Biorad) according to manufactures protocol. After protein transfer, blots were blocked in 5% BSA/TBS for 1 h and stained for SOX2 (1:100, Millipore AB5603), SOX9 (1:250; cell signaling 82630) and ß-actin (1:1000; Chemicon, C4 MAB 1501) in 5% BSA/TBS over night at 4 °C. Next day, blots were washed and stained with secondary antibodies in 5% BSA/TBS for 1 h at room temperature. After another 3 TBS washes, blots were stained with SuperSignal™ West Femto Maximum Sensitivity Substrate (ThermoFisher) and analyzed with LiCor analyser.

### Sample collection

Samples were collected at different days throughout the differentiation. First samples were taken at day 3 (D3) of protocol, 1 day after medium change towards NMM with dorsomorphin and SB431542. Second samples were taken at day 9 (D9), before rosette structures were cut, reminiscent to the early stage of secondary neurulation [26] followed by third sample collection at day 15 (D15), after rosettes were manually picked, comparable to complete neural tube formation state. Finally, fourth samples were taken at day 22 (D22), after first passage was performed, and cells were recovered. To collect, cells were washed once with PBS and then scraped off the cell culture plate. Solution was collected in an Eppendorf Microtube and centrifuged at maximum speed for 5 min. Supernatant was discarded, and pellet was frozen at – 80 °C until further processing for mass spectrometry.

### Cell lysis and protein digestion

Samples were lysed, reduced, and alkylated in lysis buffer (1% sodium deoxycholate (SDC), 10 mM tris(2-carboxyethyl)phosphine hydrochloride (TCEP), 40 mM chloroacetamide (CAA), and 100 mM TRIS, pH 8.0 supplemented with phosphatase inhibitor (PhosSTOP, Roche)) and protease inhibitor (Complete mini EDTA-free, Roche). After sonication, samples were centrifugated at 20,000×*g* for 20 min. Protein concentration was estimated by a BCA protein assay. Reduction was done with 5 mM ammonium bicarbonate and dithiothreitol (DTT) at 55 °C for 30 min followed by alkylation with 10 mM iodoacetamide for 30 min in dark. Proteins were then digested into peptides by LysC (protein-enzyme ratio 1:50) at 37 °C for 4 h and trypsin (protein-enzyme ratio 1:50) at 37 °C for 16 h. Peptides were then desalted using C18 solid phase extraction cartridges (Waters).

### Tandem mass tag (TMT) 10 plex labeling

Aliquots of ~ 100 μg of each sample were chemically labeled with TMT reagents (Thermo Fisher) according to Fig. 1. In total, three TMT mixtures were created for each biological replicate. Peptides were resuspended in 80 μl resuspension buffer containing 50 mM HEPES buffer and 12.5% acetonitrile (ACN, pH 8.5). TMT reagents (0.8 mg) were dissolved in 80 μl anhydrous ACN of which 20 μl was added to the peptides. Following incubation at room temperature for 1 h, the reaction was then quenched using 5% hydroxylamine in HEPES buffer for 15 min at room temperature. The TMT-labeled samples were pooled at 1:1 ratios followed by vacuum centrifuge to near dryness and desalting using Sep-Pak C18 cartridges.

### Off-line basic pH fractionation

Before the mass spectrometry analysis, the TMT mixture was fractionated and pooled using basic pH Reverse Phase HPLC. Samples were solubilized in buffer A (5% ACN, 10 mM ammonium bicarbonate, pH 8.0) and subjected to a 50 min linear gradient from 18 to 45% ACN in 10 mM ammonium bicarbonate pH 8 at flow rate of 0.8 ml/min. We used an Agilent 1100 pump equipped with a degasser and a photodiode array (PDA) detector and Agilent 300 Extend C18 column (5 μm particles, 4.6 mm i.d., and 20 cm in length). The peptide mixture was fractionated into 54 fractions and consolidated into 20. Samples were acidified with 10% formic acid and vacuum-dried followed by re-dissolving in 5% formic acid/5% ACN for LC-MS/MS processing.

### Mass spectrometry analysis

We used nanoflow LC-MS/MS using Orbitrap Lumos (Thermo Fisher Scientific) coupled to an Agilent 1290 HPLC system (Agilent Technologies). Trap column of 20 mm × 100 μm inner diameter (ReproSil C18, Dr Maisch GmbH, Ammerbuch, Germany) was used followed by a 40 cm × 50 μm inner diameter analytical column (ReproSil Pur C18-AQ (Dr Maisch GmbH, Ammerbuch, Germany)). Both columns were packed in-house. Trapping was done at 5 μl/min in 0.1 M acetic acid in H_2_O for 10 min, and the analytical separation was done at 300 nl/min for 2 h by increasing the concentration of 0.1 M acetic acid in 80% acetonitrile (*v*/*v*). The mass spectrometer was operated in a data-dependent mode, automatically switching between MS and MS/MS. Full-scan MS spectra were acquired in the Orbitrap from m/z 350–1500 with a resolution of 60,000 FHMW, automatic gain control (AGC) target of 200,000 and maximum injection time of 50 ms. Ten most intense precursors at a threshold above 5000 were selected with an isolation window of 1.2 Da after accumulation to a target value of 30,000 (maximum injection time was 115 ms). Fragmentation was carried out using higher-energy collisional dissociation (HCD) with collision energy of 38% and activation time of 0.1 ms. Fragment ion analysis was performed on Orbitrap with resolution of 60,000 FHMW and a low mass cut-off setting of 120 m/z.

### Data processing

Mass spectra were processed using Proteome Discover (version 2.1, Thermo Scientific). Peak list was searched using the Swissprot database (version 2014_08) with the search engine Sequest HT. The following parameters were used. Trypsin was specified as enzyme, and up to two missed cleavages were allowed. Taxonomy was set for Homo sapiens, and precursor mass tolerance was set to 50 ppm with 0.05 Da fragment ion tolerance. TMT tags on lysine residues and peptide N termini and oxidation of methionine residues were set as dynamic modifications, and carbamidomethylation on cysteine residues was set as static modification. For the reporter ion quantification, integration tolerance was set to 20 ppm with the most confident centroid method. Results were filtered to a false discovery rate (FDR) below 1%. Finally, peptides lower than 6 amino-acid residues were discarded. Within each TMT experiment, reporter intensity values were normalized by summing the values across all peptides in each channel and then corrected for each channel by having the same summed value. After that, the normalized S/N values were summed for all peptides. Finally, proteins were log_2_ transformed and normalized by median subtraction. Proteins were included that are identified in 2 out of 3 clones.

### Data visualization

The software Perseus was used for data analysis and to generate the plots. Volcano plots for each time point were generated, and up- or downregulated proteins were considered significant with a fold change cut-off = 1.3. Functional analysis to enrich to GO terms was done on David Database, and pathway enrichment analysis was done on Reactome Functional Interaction (http://www.reactome.org/). Furthermore, protein interaction network was performed using Cytoscape, Gnenmania plugin.

### Statistics

Volcano plots were generated for each time point and because of the tight ratios typically observed in TMT quantification [27], we selected a cut-off for proteins being up- or downregulated with a *p* value ≤ 0.1 and ≥ 1.3-fold change difference in RTT compared to iCTR. These cutoffs are based on the observed distribution in the volcano plot and are affected by the ratio compression observed in isobaric quantification strategies, caused by impure MS1 precursor isolation [28]. To study protein subsets consistently dysregulated at all time points, we grouped all RTT at all time points together and all iCTR at all time points together. A volcano plot was generated, and proteins were considered significantly up- or downregulated with a *p* value ≤ 0.1 and ≥ 1.3-fold change difference in RTT compared to iCTR. To measure protein abundance changes in SOX2 and SOX9 by western blot analysis, we calculated the relative intensity by normalizing the values to the loading control ACTIN using the ImageJ software. Considering normal distribution, the mean values of the three RTT samples and three iCTR samples were compared by using a standard unpaired *t* test and considered significantly different when *p* < 0.05.

## Results

### Generation of iPSC-derived neuronal progenitors from RTT and isogenic controls

**Fig. 2:**
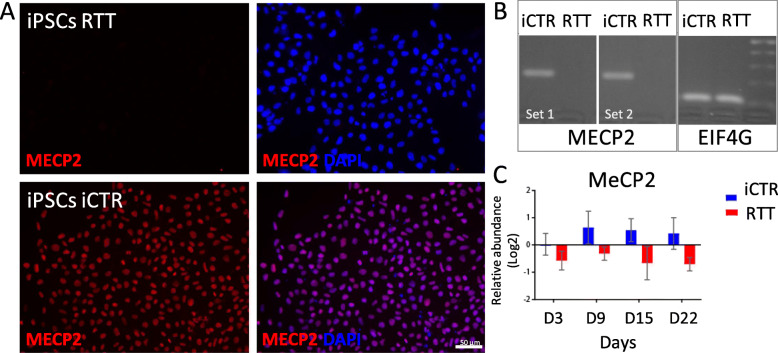
iCTR and RTT cell line validation. **a** Representative immunohistochemical staining for MeCP2 in RTT iPSC line (upper) and iCTR iPSC line (lower). **b** PCR results of iCTR and RTT iPSC lines for two different primer sets spanning deletion Del_Ex3-4 and Exon2 as positive control. **c** Relative abundance level of MeCP2 in RTT NES and iCTR NES lines at different time points of sample collection

RTT patient and iCTR fibroblasts were reprogrammed into iPSCs via electroporation of reprogramming plasmids [24]. Pluripotency was confirmed using classic assays, including immunocytochemistry (Additional file 1: Figure S1a) and RNA expression (Additional file 1: Figure S1b). Expression of mutated and healthy MeCP2 in the RTT and iCRT lines was confirmed by immunocytochemistry and PCR for *MECP2* (Fig. 2a, b). Additionally, the protein expression level of MeCP2 detected by mass spectrometry showed a higher expression of MeCP2 in iCTR relative to RTT samples (Fig. 2c, Additional file 1: Figure S1c). A detectable low expression of mutant alleles, here MeCP2 expression in mutant RTT lines, could be caused by the so-called co-isolation issue in TMT experiments [27]. Generation of NES cells was performed as described before [21]. Neuronal induction into neuronal rosette structures was monitored by visual inspection and appeared after approximately 12 days of neuronal development initiation (Fig. 1). Time points for sample collection were chosen along the differentiation towards neuronal progenitor cells.

### MS-based quantitative proteomics during neuronal development

**Fig. 3:**
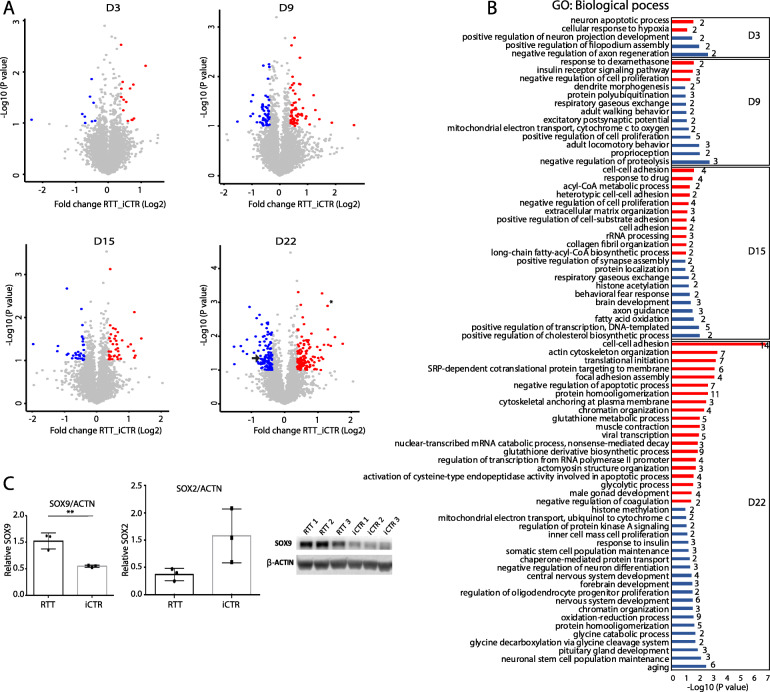
Volcano plots and GO analysis at different time points. **a** Volcano plot demonstrating proteins differentially regulated in RTT compared to iCTR at each time point of neuronal development. Each data point represents a single quantified protein. The *x*-axis represents the log2-fold change in abundance (RTT/iCTR) and *y*-axis the − log10 (*p* value). Threshold for significant proteins is chosen for *p* value cut-off (0.1) and fold change ≥ 1.3. Proteins in blue indicate for downregulation and in red indicate for upregulation in RTT. Arrow in day 22 points for SOX2 expression, and asterisk points for SOX9 expression. **b** Gene Ontology analysis of the significant proteins on their biological function at each time point of neuronal differentiation. Red indicates the upregulated and blue the downregulated biological processes. The numbers indicate the genes enriched for each term. **c** Western blot analysis showing SOX9 and SOX2 expression in iCTR NES lines at D22. Significant increase in SOX9 expression in RTT samples, and SOX2 shows a trend towards decrease in RTT samples

To study proteomic changes between RTT and iCTR during neuronal development, cell lysates at indicated time points were subjected to tryptic digestion, high-pH fractionation followed by high-resolution tandem mass spectrometry (LC-MS/MS) analysis and TMT-10plex quantification (Fig. 1). In total, we identified up to 7702 proteins, of which 3658 proteins were identified in all samples (Additional file 1: Figure S2, Table S1). Next, to determine protein expression changes over the time points, we compared RTT versus iCTR and considered proteins with a *p* value ≤ 0.1 and a fold change ≥ 1.3 in 2 out of 3 clones as significantly regulated (Fig. 3a, Table S2). This resulted in 23 up- or downregulated proteins at D3, 111 at D9, 72 at D15, and 243 at D22, between RTT and iCTR. We then compared the overlap of these regulated proteins across the different time points, which we presented in a Venn diagram (Additional file 1: Figure S3). We noticed that the majority of the regulated proteins only have 0–2% overlap between the different time points. To better understand what biological processes are involved at each time point, we performed Gene Ontology (GO) enrichment analysis with respect to biological functions (Fig. 3b). GO analysis on the significantly upregulated proteins in RTT versus iCTR revealed the GO terms *neuron apoptotic process* and *cellular response to hypoxia* at D3. GO terms related to downregulated proteins in RTT at D3 involved *negative regulation of axon regeneration*, *positive regulation of filopodium assembly*, and *positive regulation of neuron projection development*. At D9, *insulin receptor signaling pathway* was upregulated whereas *excitatory postsynaptic potential* was downregulated in RTT. At D15, *cell-cell adhesion* and *acyl-CoA metabolic process* were upregulated, and terms such as *axon guidance*, *brain development*, and *histone acetylation* were downregulated. Furthermore, at D22, GO terms related to *cell-cell adhesion* and *actin cytoskeleton organization* were upregulated, and *nervous system development* and *forebrain development* were downregulated in RTT. Terms such as *brain development* were downregulated in D15 as well as D22. Gene set enrichment analysis (GSEA) of all up- and downregulated proteins further revealed that RTT-associated proteins were strongly enriched in gene sets such as *apoptosis*, *DNA repair*, and in *metabolism* (Additional file 1: Figure S4, Table S3). To further verify the results of the mass spectrometry, we performed western blot analysis for proteins SOX2 and SOX9, transcription factors with pivotal role in development and differentiation [29, 30], which showed significant differences in expression levels between iCTR and RTT lines at D22 (Fig. 3a, c). In line with mass spectrometry data, western blot analysis showed a significant increase in SOX9 expression levels in RTT lines when compared to iCTR (*p =* 0.0057, unpaired *t* test), and a decrease in SOX2 expression in RTT lines at D22, although this did not reach statistical significance (*p =* 0.07, unpaired *t* test). Together, both approaches demonstrate that SOX2 and SOX9 were differentially expressed between RTT and iCTR, thereby validating our findings that RTT samples show proteome changes at early neurodevelopmental changes

A previous study identified perturbed astrocyte differentiation of RTT-iPSCs, suggesting skewed differentiation of neural progenitor cells into neuronal cell lineage [31]. Here, we studied whether we could find similar changes, i.e., increased neuronal marker MAP2 and decreased glia marker (e.g., ATP1A2, CLU, and SLC1A3) expression. While these astrocyte and neuronal markers are expressed in our samples, we found no significant differences between iCTR and RTT samples (Additional file 1: Figure S3). Furthermore, while the authors showed higher expression of LIN28 in RTT samples, we identified two isoforms (LIN28A and B) with no differences between RTT and iCTR samples. In order to evaluate whether the altered proteomes are present in the human brain, we compared our proteomic altered data to the Allen Brain atlas. This is showing individual gene expression in the different brain areas (Supplementary Table S4). This revealed high variability of expression between transcriptomics and proteomics. The majority of the proteins have measurable expression levels in human brain in vivo. Overall, we show that proteins associated with neuronal development are differentially expressed in RTT at early stages of neuronal differentiation.

### Coordinated proteome alteration during neuronal development in RTT syndrome

**Fig. 4:**
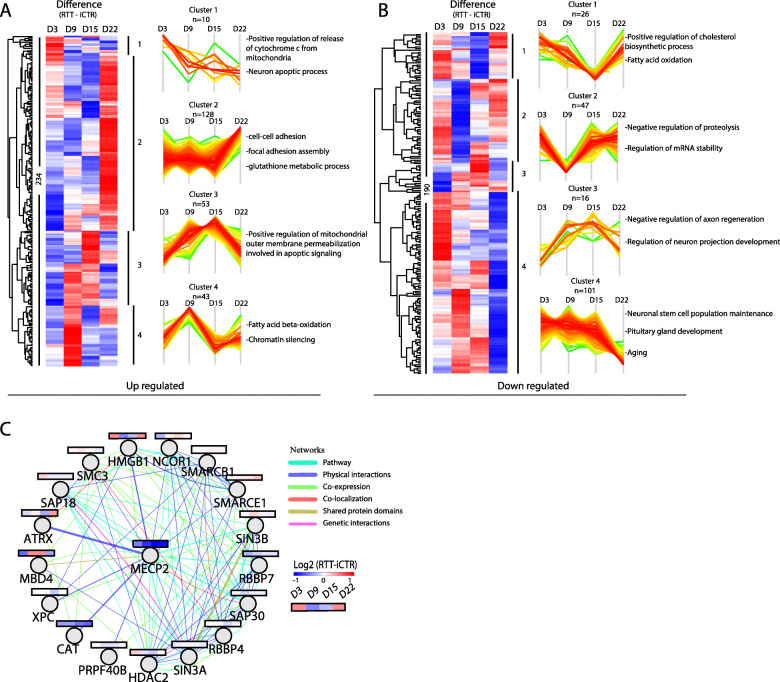
Up- and downregulated proteins and MeCP2-binding partners. **a** Heat map of all significant up- and **b** downregulated proteins with a *p* value ≤ 0.1 and ≥ 1.3-fold change between RTT and iCTR. The *Z* score of the difference between RTT and iCTR is given for each day with the corresponding cluster analysis and the GO terms for biological processes. **c** Network analysis of MeCP2-binding proteins identified in our data. The average log2 ratio RTT-iCTR over time is color coded for each protein over the time course of neuronal development. Edges are color coded according to the network as indicated

To gain insight into how the differentially expressed proteins in RTT behave across time points, we further analyzed all the significantly up- or downregulated proteins at D3, D9, D15, and D22. This resulted in 234 significantly up and 190 downregulated proteins. The average log2 values of RTT were extracted with iCTR for each time point, and the difference between RTT and iCTR is shown in a heat map (Fig. 4a). To obtain an unbiased view of the differentially expressed proteins during neuronal differentiation, we performed cluster analysis on the significantly up- or downregulated proteins. This resulted in four clusters for both the up- or downregulated proteins with distinct expression profiles. Cluster 1 contains proteins strongly upregulated in D3 that are involved in *neuron apoptotic processes* and *cytochrome c release from mitochondria*-related GO terms. Cluster 2 represents upregulated proteins at D22 that are involved in *adhesion assembly* and *glutathione metabolic processes*. Cluster 3 contains proteins upregulated at D9 and D15 having a role in *apoptotic signaling*, and cluster 4 represents proteins strongly upregulated at D9 having a role in *oxidation* and *chromatin silencing*. In the downregulated proteins, cluster 1 represents proteins that showed a strong downregulation at D15 mainly involved in *cholesterol biosynthesis* and *fatty acid oxidation* (Fig. 4b). Cluster 2 represents proteins strongly downregulated at D9, which are associated with *regulation of proteolysis* and *mRNA stability*. Cluster 3 of the downregulated proteins in RTT shows a decrease expression profile in D3 which are involved in *axon regeneration* and *neuron projection development*, and cluster 4 covers many proteins strongly downregulated in D22 involved in processes such as *neuronal stem cell maintenance*, *pituitary gland development*, and *aging*. Collectively, our data reveals a changing, stage-specific pattern of the differentially expressed proteins during neuronal development in RTT.

### MeCP2 network analysis

To further investigate the proteins that are targets by the MeCP2 protein, we drew a protein interaction network (Cytoscape, Genemania plugin) using MeCP2 protein as input (Fig. 4c). The data covered 20 MeCP2-interacting proteins of which 18 proteins were identified in our data. To further investigate how the MeCP2- interacting proteins change over time in RTT, we extracted the average log2 values of iCTR by RTT samples to represents the difference at each time point. As expected, MeCP2 is downregulated along the course of neuronal differentiation in RTT samples compared to iCTR. The proteins are tightly interconnected around HDAC2, SIN3A, RBBP4, SAP30, SMARCE1, SMARCB1, and MeCP2 but to a lesser extend around CAT, XPC, PRPF40B, and MBD4. The network revealed several RNA/DNA binding proteins of which MeCP2 and CAT are one of the most downregulated proteins in RTT. While some proteins in the network, such as SMARCB1 and SIN3A, stayed constant over time, others showed changing levels, such as MBD4 and HMGB1. Interestingly, MBD4, next to MeCP2, is a member of the methyl-CpG-binding domain (MBD) family. Overall, we searched for MeCP2-binding partners and showed how these proteins change along the course of neuronal differentiation in RTT and iCTR.

### Protein subsets consistently dysregulated at all time points

**Fig. 5:**
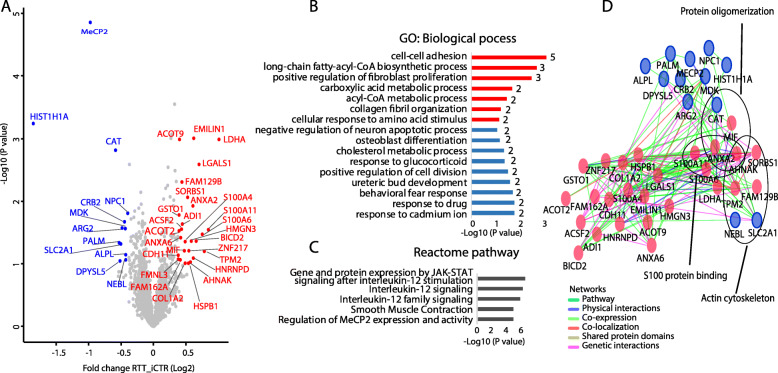
Volcano plot all days and network analysis. **a** Volcano plot demonstrating proteins differentially expressed in RTT versus iCTR after pooling all time points of neuronal development. The *x*-axis represents the log2-fold change in abundance (RTT/iCTR) and *y*-axis the − log10 (*p* value). Threshold for significant proteins is chosen for *p* value cutt-off (0.1) and fold change ≤ 1.3. Upregulated proteins in RTT are shown in red, and downregulated are shown in blue. **b** GO analysis on the biological process of the significant proteins. *X*-axis represents the − log10 (*p* value), and red and blue colors indicate for up- and downregulated proteins in RTT, respectively. The numbers represent the genes enriched for each term. **c** Reactome pathway analysis of the significant proteins. **d** Network analysis of the significant proteins by Cytoscape plugin GeneMania. Red indicates for upregulated, and blue indicates for downregulated proteins in RTT

To study proteins differentially expressed between RTT and iCTR regardless the time point of differentiation, we grouped all RTT and all iCTR samples from all time points together. Due to the tight ratios typically observed in TMT quantification [27], we selected a cut-off for proteins being up- or downregulated with a *p* value ≤ 0.1 and ≥ 1.3-fold change difference in RTT compared to iCTR based on the observed distribution in the volcano plot (Fig. 5a). We identified 27 proteins being up and 12 proteins being downregulated in RTT compared to iCTR. As expected, MeCP2 was one of the most strongly downregulated proteins in RTT. GO analysis revealed biological processes such as *cell-cell adhesion* and *acyl-CoA metabolic processes* to be upregulated (Fig. 5b), which are also upregulated at individual time points D15 and D22 (Fig. 4a). In contrast, several processes such as *response to cadmium ion*, *response to drug*, and *behavioral fear response* were downregulated in RTT (Fig. 5b). Analysis of the differentially regulated proteins using Reactome pathway analysis revealed among others, *JAK/STAT signaling after Interleukin-12 stimulation* and *regulation of MeCP2 expression and activity* to be differentially expressed in RTT versus iCTR (Fig. 5c). To further visualize the connectivity among these significant proteins, we analyzed their protein networks in the Cytoscape tool (Genemania plugin). A high degree of connectivity, such as being co-expressed and having shared genetic interactions, around these proteins was identified. Interestingly, the majority of the proteins are involved in immunity, actin cytoskeleton organization, and calcium binding (Fig. 5d). Together, we show that proteins associated with immunity and metabolic processes are differentially expressed in RTT in a time-point independent manner during differentiation towards neuronal progenitors.

## Discussion

To gain insight into mechanisms involved in early neurodevelopmental changes in RTT patients with MeCP2 deficiency, we performed mass spectrometry-based quantitative proteomic analysis on samples derived from RTT patient and isogenic control iPSCs at days 3, 9, 15, and 22 of neuronal induction. We identified altered proteins associated to multiple pathways, of which *cell-cell adhesion* and *actin cytoskeleton organization* show most significant upregulations, and *pituitary gland development* and *neuronal stem cell population maintenance* show most significant downregulations at D22. At D3, D9, and D15, other and less dysregulated pathways were found, including *neuron apoptotic process* and *negative regulation of axon regeneration* (D3), *response to dexamethasone* and *negative regulation of proteolysis* (D9), and *cell-cell adhesion* and *positive regulation of cholesterol biosynthetic process* (D15) (Fig. 3). The number of altered proteins, as well as an increase in the fold change of protein alteration in RTT samples, gradually increased from day 3 to day 22. However, at all time points, altered pathways associated to neurodevelopment or neurogenesis with at least a 1.3-fold up- or downregulation, could be identified in RTT samples. This report shows that exons 3–4 deletion in *MECP2* results in protein expression changes at neuronal progenitor stages and provides a resource of proteins and pathways for further exploration to identify common disease mechanisms underlying RTT and related phenotypes.

### RTT samples present protein expression changes that become more apparent from early to late neuronal progenitor stages

The altered proteins in the RTT cultures gradually increased from D3 to D22 samples. As RTT patients start to show disease-related clinical symptoms at 6–18 months of age, it is likely molecular changes already start at prenatal stages and progress over time [8]. Based on mouse studies, expression of MeCP2 is already present during early embryogenesis [32] and gradually increases at later stages, suggesting its progressive importance in brain development [14, 33]. In line with this, it is expected that lack of MeCP2 will increasingly affect gene regulation and so the number of altered proteins, which converge and lead to clinical manifestation. While not confirmed on protein levels, transcriptional alterations in RTT samples during early neuronal developmental stages have been shown [34, 35]. Changes in the transcriptome were already observed at the pluripotent stem cell stage when iPSCs of RTT patients were compared to human embryonic stem cells [36]. Here, we identified alterations on protein levels occurring only 3 days after neuronal induction of pluripotent stem cells. This time point is comparable to a brain developmental stage of 3 months of gestational age [37]. So, our study supports the hypothesis of an early post gestational onset of molecular changes, leading to clinical phenotype at early postnatal stages.

### Protein expression changes in neuronal progenitor cells from RTT patients

The most significant upregulated proteins are associated with GO terms *cell-cell adhesion* and *actin cytoskeleton organization*. In previous studies, dysregulation of the actin cytoskeletal organization was observed on transcriptional level and associated with phenotypic alterations as abnormal dendrite formation and dendritic growth [38–41]. However, these studies reported a transcriptional downregulation of the mentioned pathways, which is in contrast to our results. Due to the poor correlation of transcriptome and proteome [42] as well as the early developmental time points in our study, different effects on the named pathways can be assumed. As specifically actin play a very dynamic role in neurodevelopment, it is important to compare samples with regard to their developmental stage [43, 44]. On the other hand, the downregulation of proteins in pathway *neuronal stem cell population maintenance* we found was observed in mouse cells before, showing a reduced proliferation rate of neuronal stem cells as well as alterations in neuronal development [45]*.* Also, we found downregulated pathways indicative of affected *nervous system development* and *forebrain development*, which is associated with the typical neuronal phenotype in RTT. Reduced dendritic outgrowth and decreased spine density has been shown in RTT mouse models [46–49] as well as neuronal morphologies typical for juvenile brains in RTT postmortem tissue [50–52]. Interestingly, already at day 3 and day 9 of neuronal induction we identified downregulation of the pathways *dendrite morphogenesis* and *axon guidance*, which are associated with specific morphological alterations observed in postmortem tissue from RTT patients [47, 50, 51]. The second most downregulated pathway in our study *pituitary gland development* was also associated with RTT before [53]. In patients with RTT syndrome, pathologist identified a reduced size of the pituitary gland [53] and dysregulation of thyroid hormone release [54]. Interestingly, one of the significantly upregulated protein at D22 was AUTS2 (Supplementary Table S2), which was found before in a MeCP2-deficient mouse model at postnatal day 60 on transcriptomic and proteomic level. This is an autism susceptibility gene implicated in various neurological disorders such as autism spectrum disorder, which has similar disease symptoms as RTT syndrome. Interestingly, all these alterations found at early neurodevelopmental stages are associated to RTT phenotypes observed at later stages.

Worth mentioning are proteins, which levels, are also dysregulated in other neurodevelopmental disorders, which patients show an overlap in neurological dysfunctions of RTT patients. We found changed levels in DOCK6 (at D3) [55, 56], NPC1 (at D9) [57, 58], CDK5 (at D9) [59, 60], HINT1 (at D9) [61, 62], CSTB (at D9) [63], ACSL4 (at D22) [64, 65], KIF5C (at D22) [66, 67], and TUBB2A (at D22) [68], which are respectively dysregulated in epilepsy, Niemann-Pick disease, mental retardation, cortical dysplasia, lissencephaly, neurotonia, and axonal neuropathy. Even though these candidate proteins were not associated with RTT before, these could be key in affected neuronal development in RTT and other neurological disorders, and therefore could be of special interest in identifying new disease mechanisms. Overall, our findings suggest that dysregulation of MeCP2 affects protein expression associated with neurodevelopmental functions at early stages of neuronal differentiation. Reported protein changes provide starting points for further research, especially those that have been identified in other neurodevelopmental disorders that present similar phenotypes.

### Expression changes of MeCP2-interacting proteins in RTT and iCTR

Network analysis using GeneMANIA [69] revealed part of these proteins to be interacting with MeCP2. Interestingly, of these interacting proteins, MBD4 is a member of the MBD family of proteins together with MeCP2. Mutations in these functional important domains tend to cause RTT-associated phenotypes [70–72]. Furthermore, HMGB1, which was downregulated in D9 and D15 in our data, was previously shown to be lower expressed in hippocampal granule neurons of Mecp2 KO mice [7].

### Protein profile changes consistently dysregulated at all time points of neuronal differentiation

When all RTT versus iCTR samples from different time points were pooled, data revealed differentially expressed proteins in RTT involved in immunity, calcium binding, and metabolism. This analysis allowed us to compensate for limited amount of biological replicates in such high-throughput technology. Several proteins associated with metabolic processes were differentially expressed in RTT, including ACSF2, ACOT2, ACOT9, LDHA, MIF, NPC1, CAT, and GSTO1. Recent evidence indicated there is perturbed lipid metabolism in RTT based on findings in brain and peripheral patient tissue, as well as metabolic dysfunctions, based on mouse studies, supporting a role for these pathways in RTT phenotypes [40, 73–76]. A previous study involving brain extracts of mice heterogeneous for MeCP2 demonstrated that disturbances in the metabolism produces changes in the morphological and biochemical development of the brains during early brain formation [74]. Another study further observed that models with synaptic defects during development fail to couple to metabolic pathways by using human independent databases [75]. Also, others earlier reported on upregulated *GSTO1* transcript levels in RTT patients’ lymphocytes together with several other mitochondrial-related genes [76]. These observations point towards involvement of the metabolic pathway in affected neuronal development.

Furthermore, our results indicate dysregulated proteins associated with immunological processes as interleuking-12 signaling. Interestingly, several publications have indicated a potential association of the immune system with MeCP2 dysregulation [77–79]. For instance, children with *MECP2* duplication show immunological abnormalities and suppressed IFN-ϒ [77]. Others showed that MeCP2 deficient patients, as well as CDKL5-related RTT patients, show dysregulated cytokine release suggestive of defective regulation of inflammatory responses [78]. Further, microglia have been proposed as key cell type in affected neurodevelopment in RTT [79]. Together, the identification of altered interleuking-12 signaling in our study supports involvement of the immune system in RTT phenotypes.

Lastly, our findings suggest changes in the calcium homeostasis in RTT, as several proteins associated with calcium signaling were altered in RTT samples. Disturbances in calcium homeostasis during early postnatal development were reported before in *Mecp2* knockout model and were suggested to play a major role in disturbed neuronal signaling in RTT [34, 80]. Based on iPSC research, the authors identified alterations in calcium signaling in RTT neurons indicative of an immature phenotype [40]. Also, others suggested involvement of calcium homeostatis in affected neuronal maturation in RTT [81]. Therefore, our findings confirm calcium signaling involvement in RTT phenotypes that already manifest at neuronal progenitor cell stages.

Altogether, our findings show pathway involvement associated to immunity, calcium signaling, and metabolism, in line with earlier studies, but now confirmed at proteome level and at much earlier developmental stages than indicated before.

## Limitations

A limitation of our study is the investigation of cells from only one donor. Although three different iPSC RTT and three isogenic control clones were included, findings should be validated in other RTT patient lines to suggest common disease mechanisms. At present, our in-depth comprehensive approach was only possible with the current number of replicates. A follow-up study with more patients might be more feasible by focusing on a few target proteins.

## Conclusion

Already at neuronal progenitor cell stages, RTT patient cells show altered protein expression levels. While both RTT patients and mouse models of RTT already show abnormalities before typical RTT-associated symptoms appear [82, 83], others never reported on neuronal phenotype specific protein changes at such early neurodevelopmental cell stages. Much of our understanding of how MeCP2 deficiency contributes to RTT disease is derived from genomic and transcriptomic studies. So far, only a few proteomic studies have been performed involving RTT human derived tissue [19, 84, 85]. The current study provides mass spectrometry-based quantitative proteomic data, depth of 7702 proteins, using an earlier developed iPSC-based models involving RTT patient and isogenic control cells [24]. We showed most significant changes in pathways associated with *cell-cell adhesion* and *actin cytoskeleton organization* as well as *neuronal stem cell population maintenance* and *pituitary gland development* and identified changes in protein expression associated with dendrite morphology or synaptic defects, previously associated with RTT [22, 35]. All these alterations were identified only shortly after neuronal induction, much earlier than actual clinical phenotypes appear. Proteins involved in immunity and metabolism, also in line with previous studies on RTT pathology [49, 73], are consistently differentially expressed. Insight into the set of altered signaling pathways or proteins, as found in this study, could support identification of underlying disease mechanisms to guide better understanding of disease phenotypes. Presented results highlight the early pre-natal onset of RTT and deserve further study.

## Supplementary information

**Additional file 1: Figure S1.** iPSC characterisation. **a**. Exemplary characterisation of iPSC lines. Immunocytochemistry for pluripotency marker (OCT3/4, SOX2, SSEA4, TRA1-60, TRA1-81). **b**. PCR-analysis for pluripotency marker. **c**. MeCP2 expression of the three iCTR and RTT samples at each time point.

**Additional file 2: Figure S2.** Number of proteins identified. A bar chart showing the number of proteins identified in each biological replicate and time point.

**Additional file 3: Figure S3.** Venn diagram. **a**. Number of proteins decreased expressed in RTT at different time points. **b**. Number of proteins increased expressed in RTT at different time points. **c**. Overview of the number of proteins altered in RTT.

**Additional file 4: Figure S4.** Top gene sets enriched in RTT-iPSCs. Proteins below p=0.1 are ranked by GSEA based on their differential expression level. Black vertical lines indicate the position where members of a pathway appear in the ranked gene list.

## Data Availability

All mass spectrometry proteomics data have been deposited to the ProteomeXchange Consortium via the PRIDE partner repository with the dataset identifier PXD013327 Username: reviewer46083@ebi.ac.uk Password: ABIw2h3I

## Abbreviations

iPSC: Induced pluripotent stem cell
MeCP2: Methyl-CpG binding protein 2
RTT: Rett syndrome
iCTR: Isogenic controls
GO: Gene Ontology
TMT: Tandem mass tag
