# Supplementary figures and images for: Quantitative proteomic analysis of Rett iPSC-derived neuronal progenitors
Source: Mol Autism. 2020 May 27;11:38. doi: 10.1186/s13229-020-00344-3 (PMC7251722; doi:10.1186/s13229-020-00344-3)

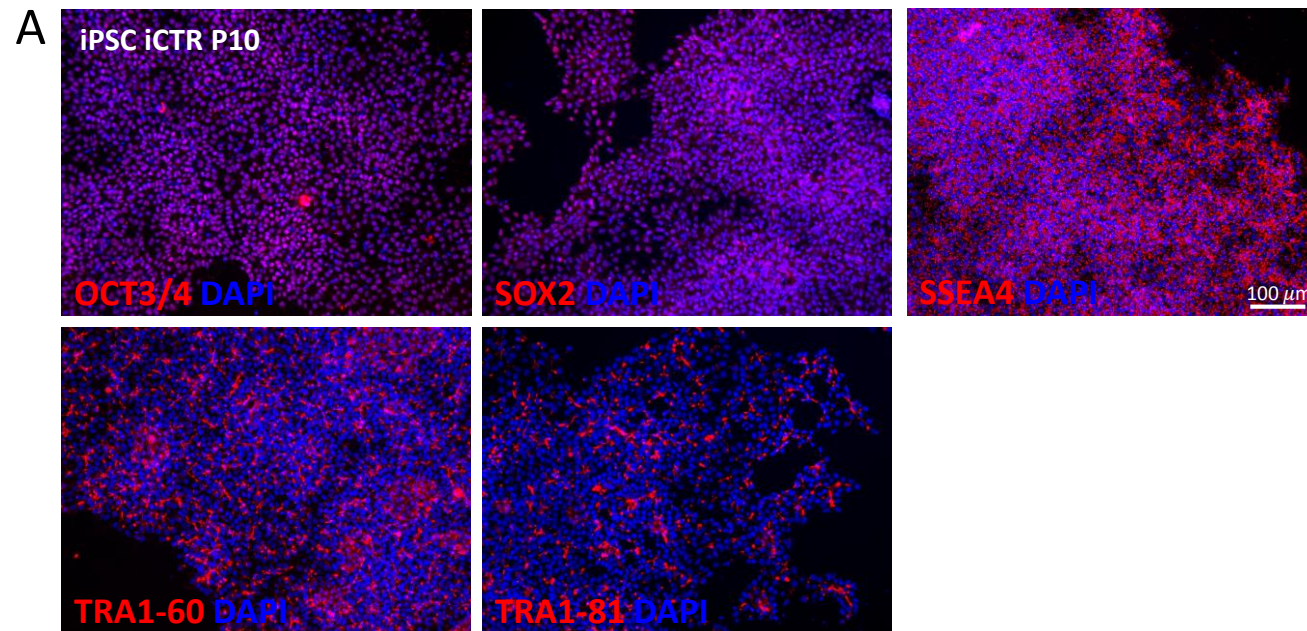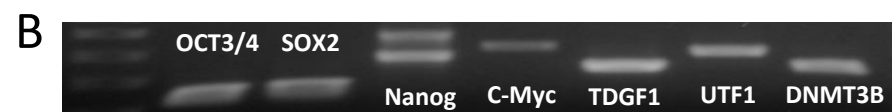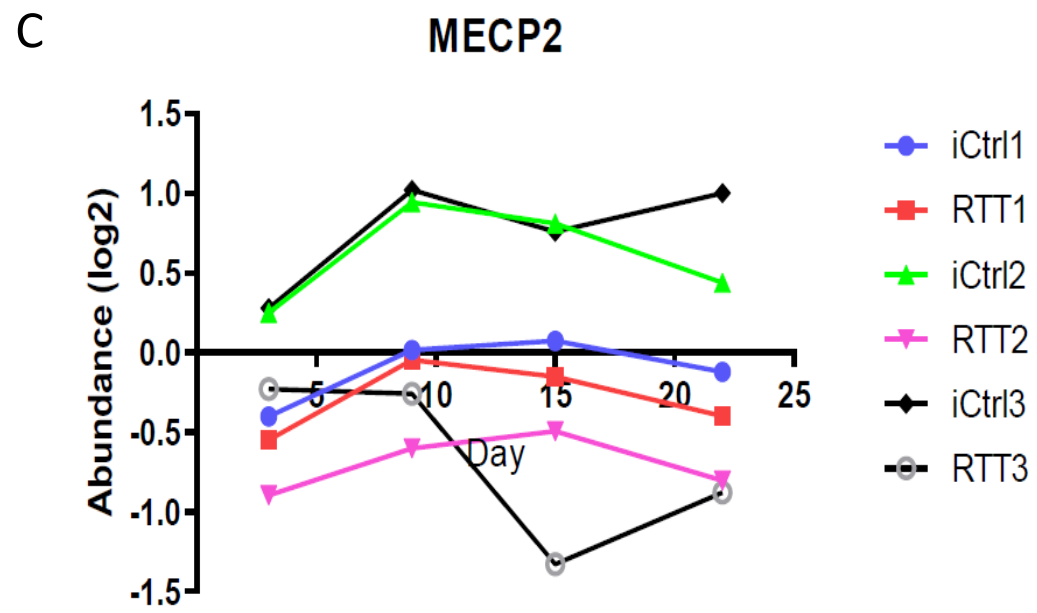

Supplement: Supplementary file 1 — Additional file 1: Figure S1. iPSC characterisation. a. Exemplary characterisation of iPSC lines. Immunocytochemistry for pluripotency marker (OCT3/4, SOX2, SSEA4, TRA1-60, TRA1-81). b. PCR-analysis for pluripotency marker. c. MeCP2 expression of the three iCTR and RTT samples at each time point. [file 13229_2020_344_MOESM1_ESM.pdf]

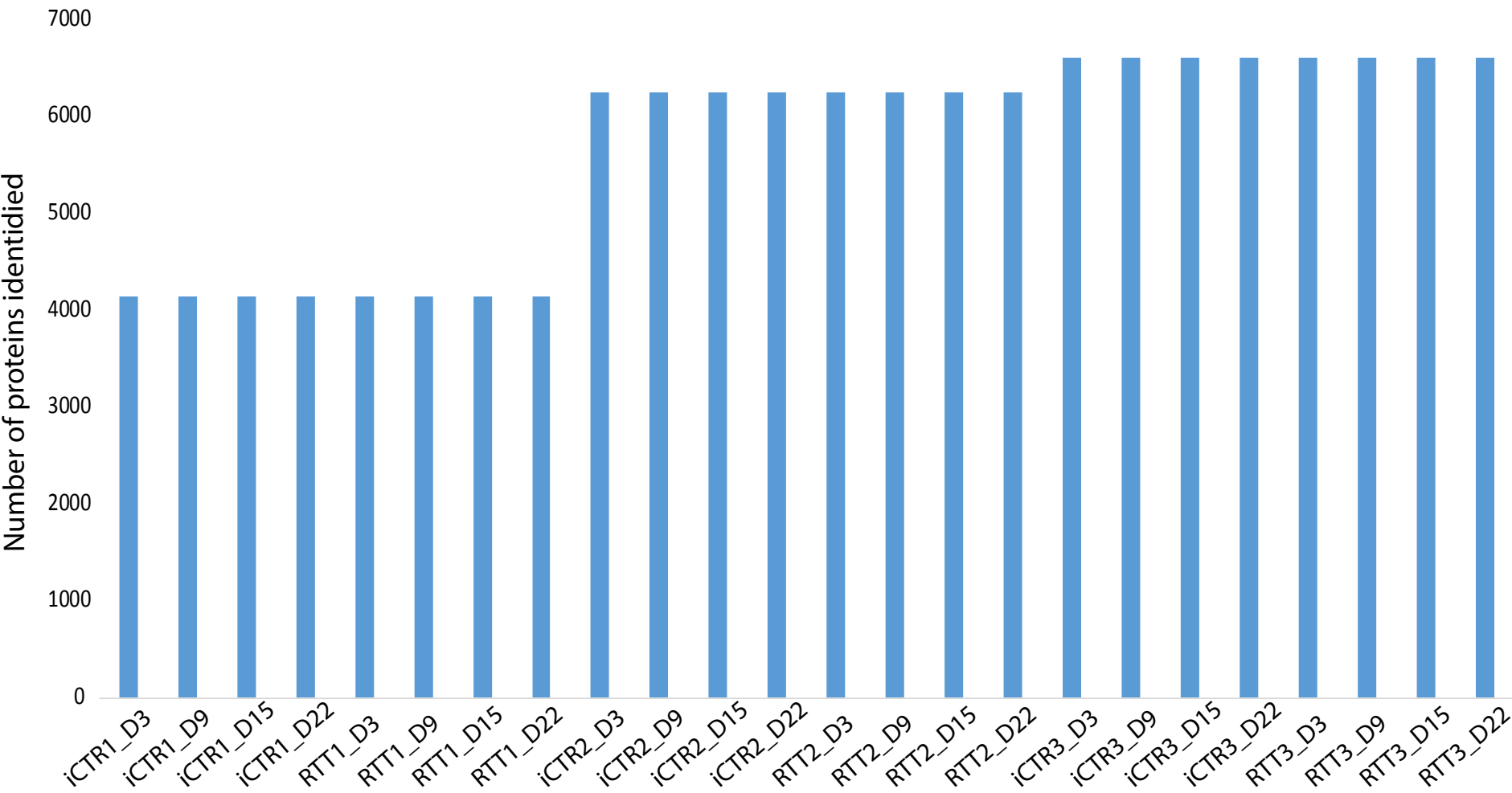

Supplement: Supplementary file 2 — Additional file 2: Figure S2. Number of proteins identified. A bar chart showing the number of proteins identified in each biological replicate and time point. [file 13229_2020_344_MOESM2_ESM.pdf]

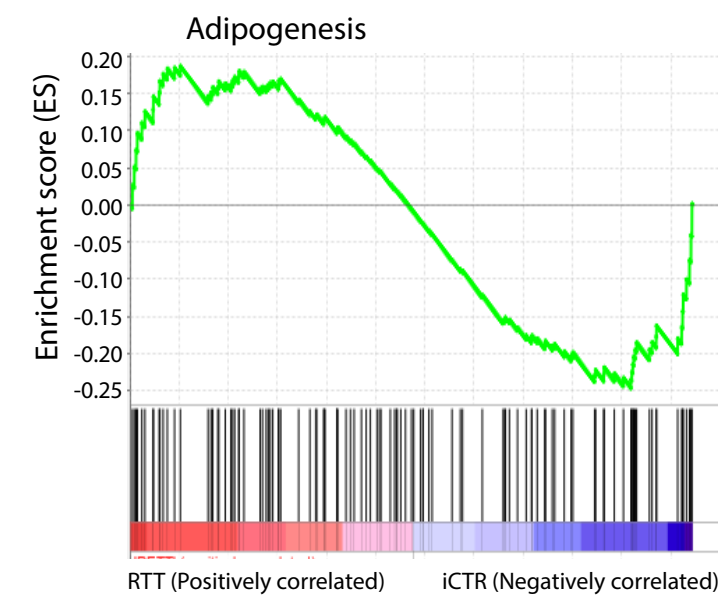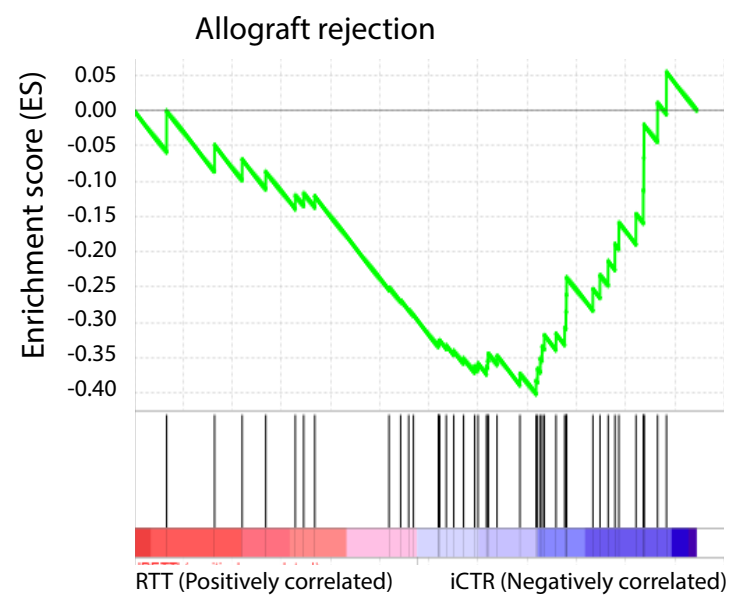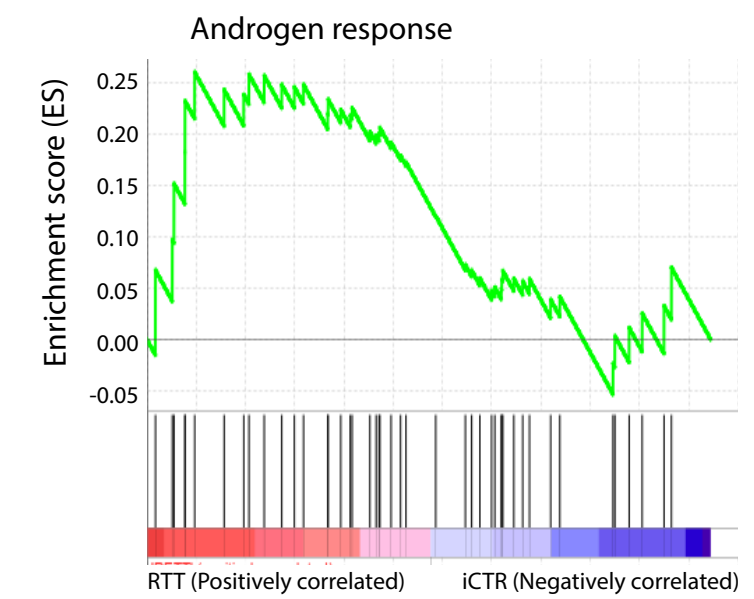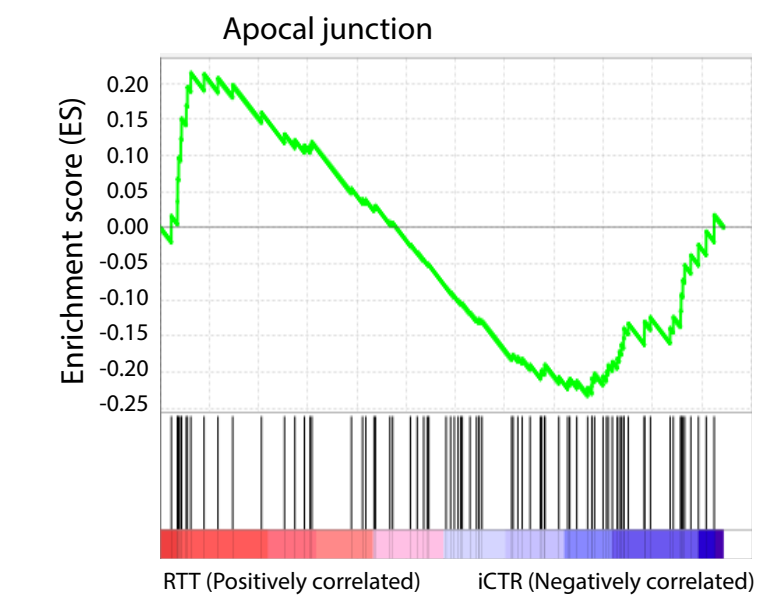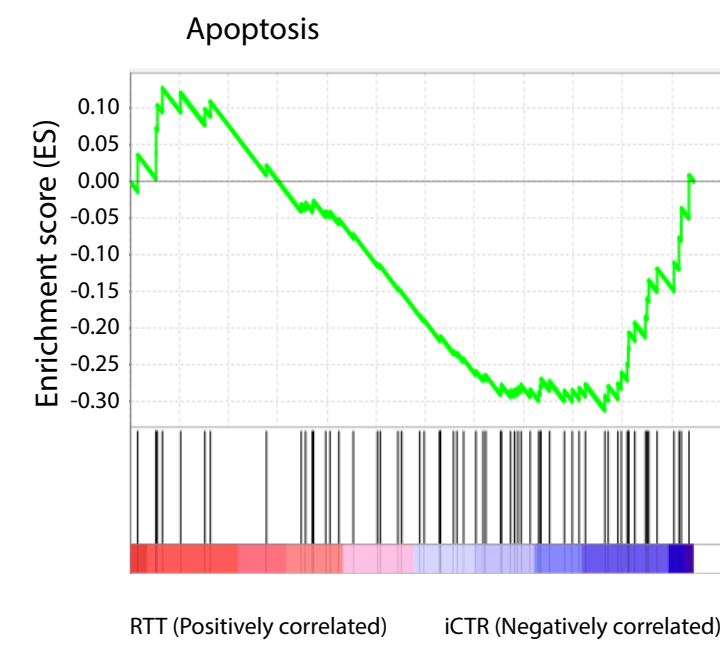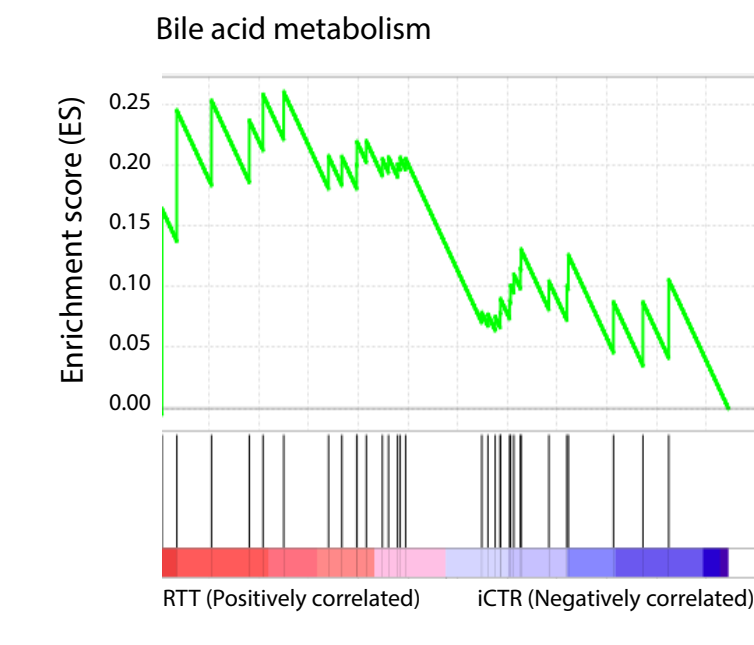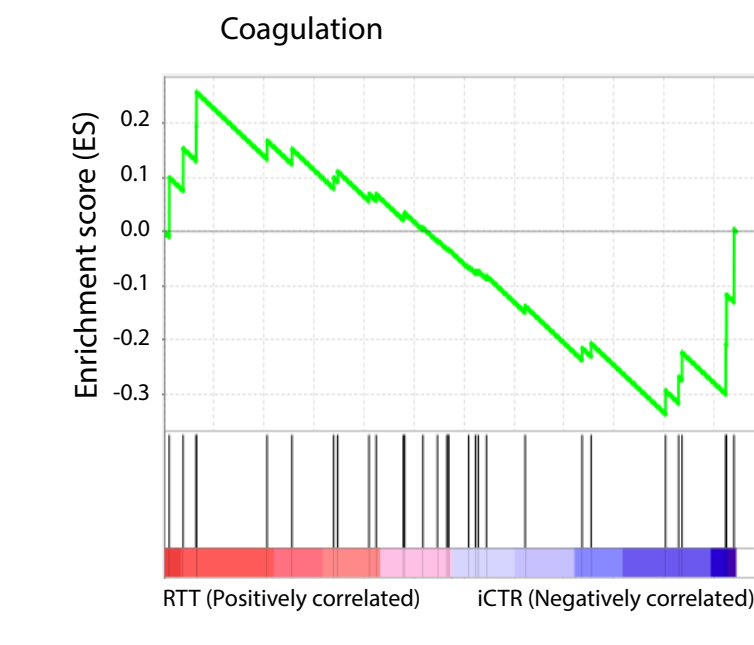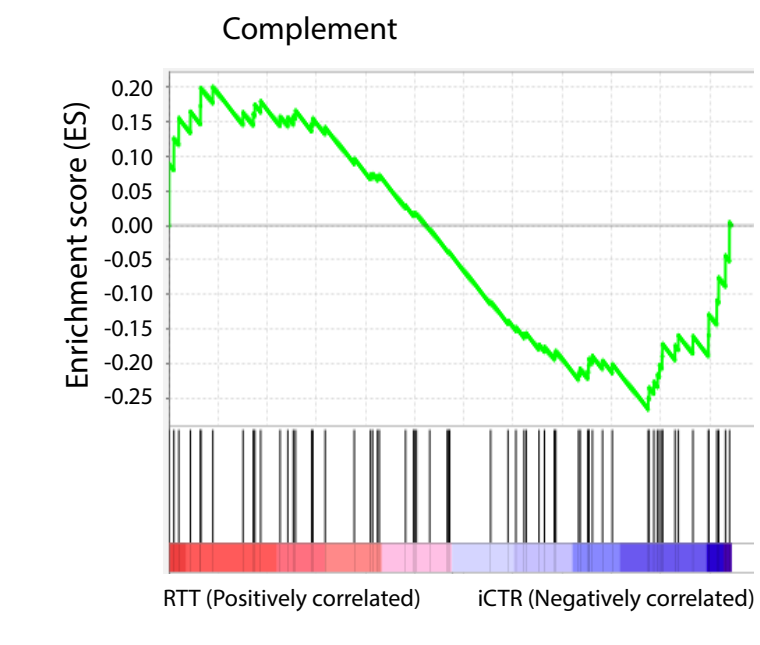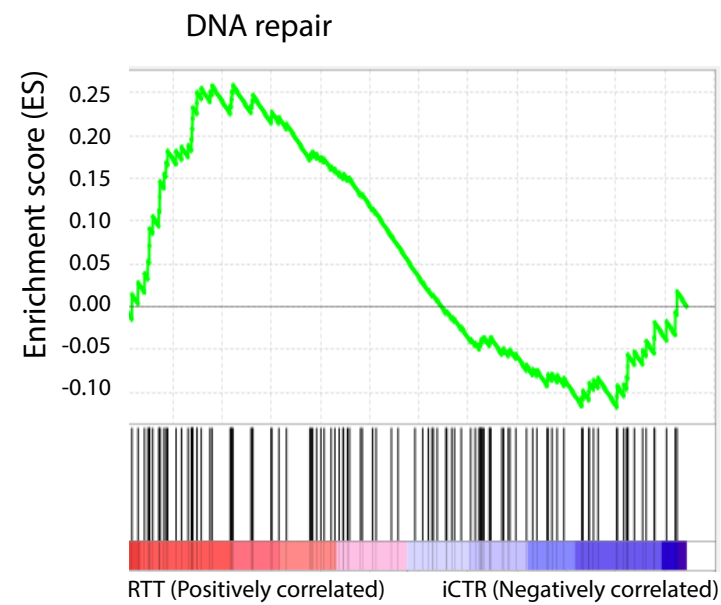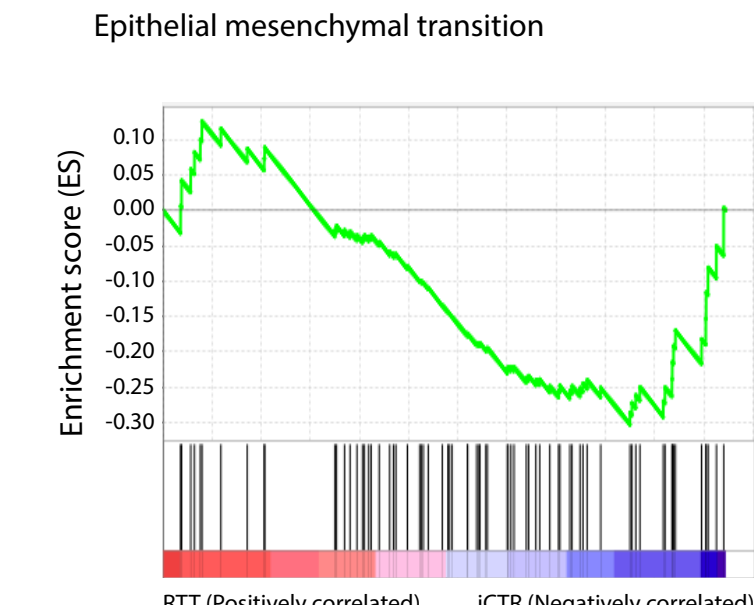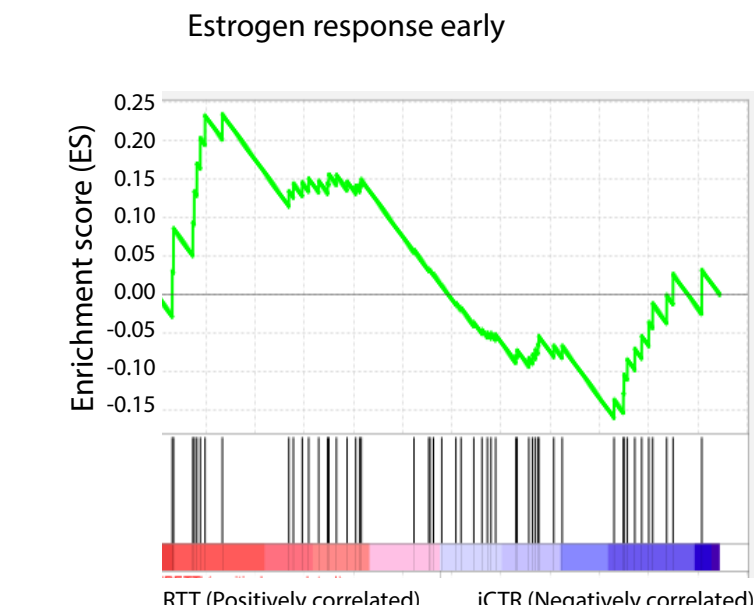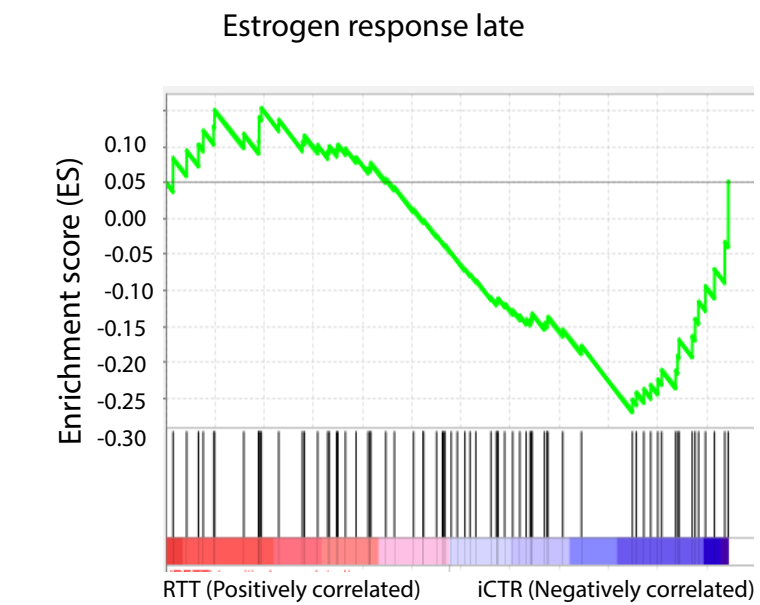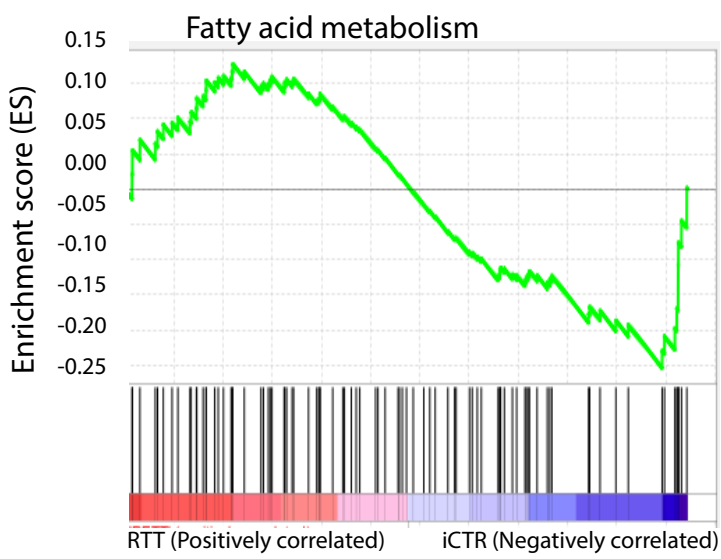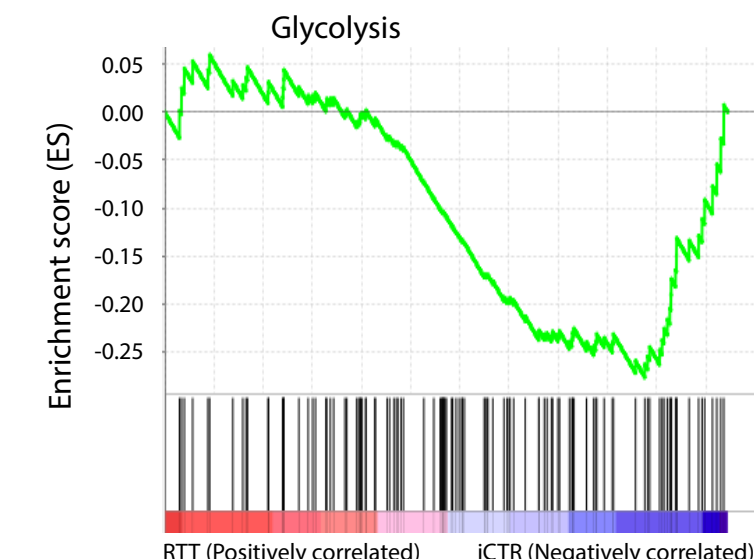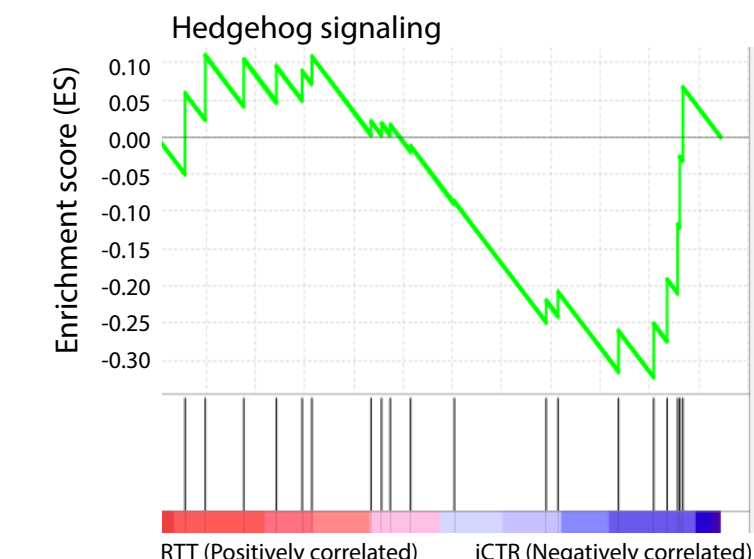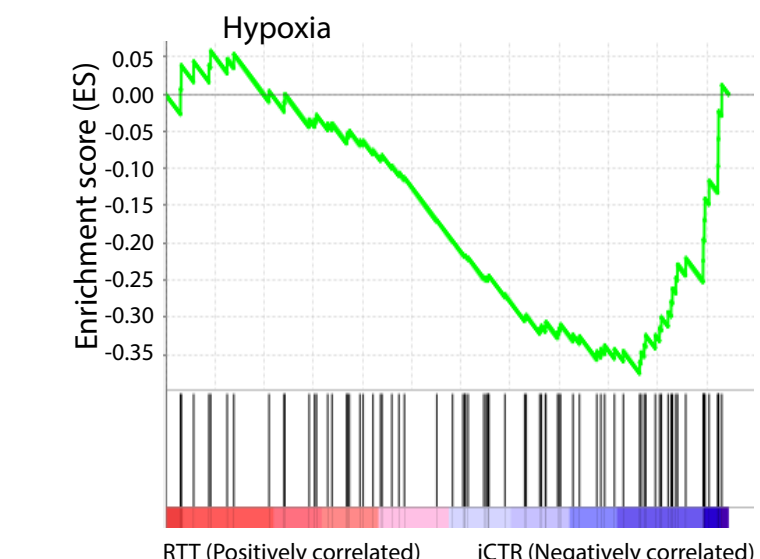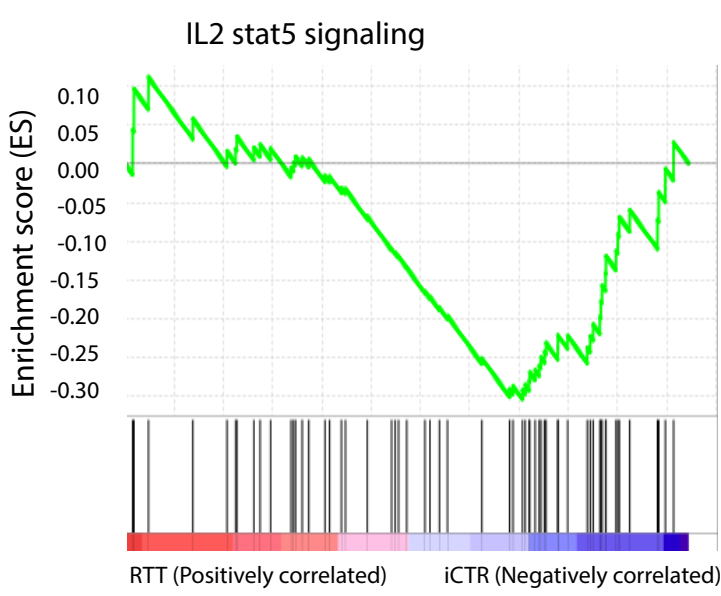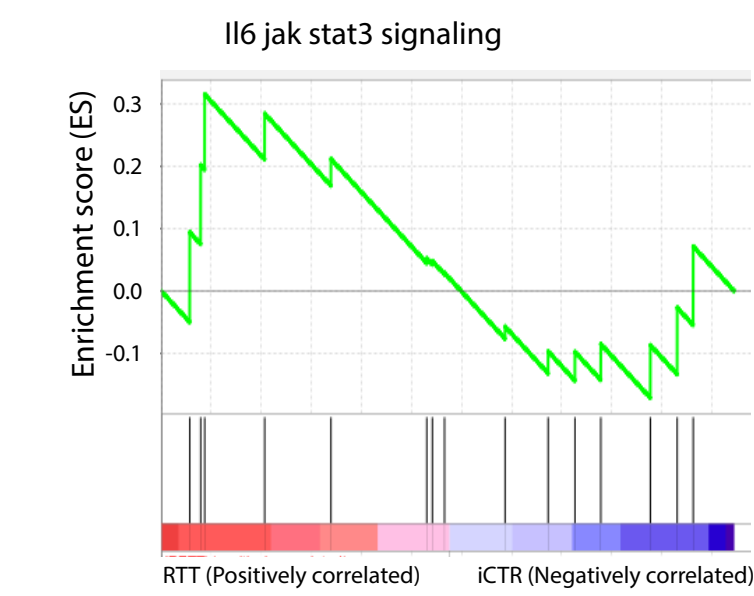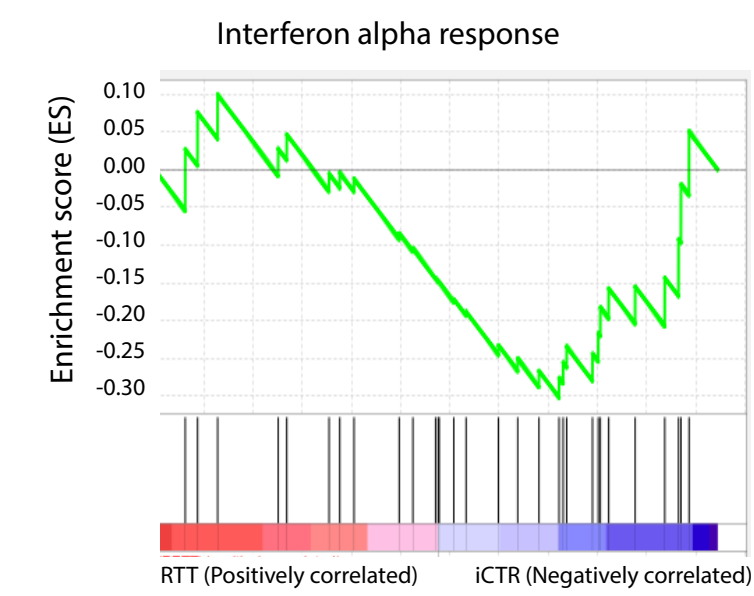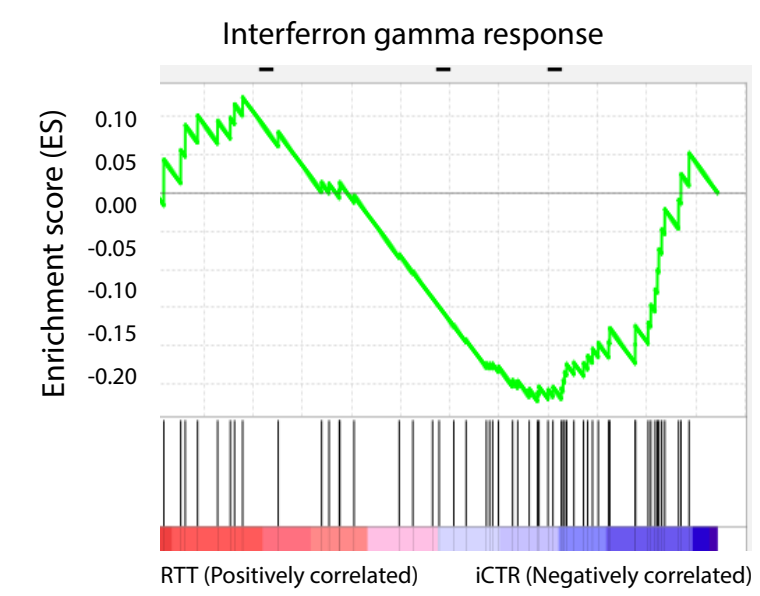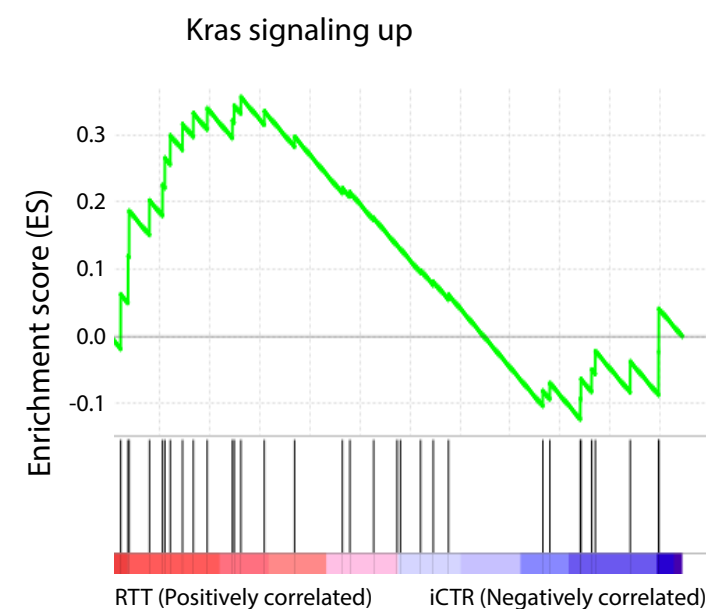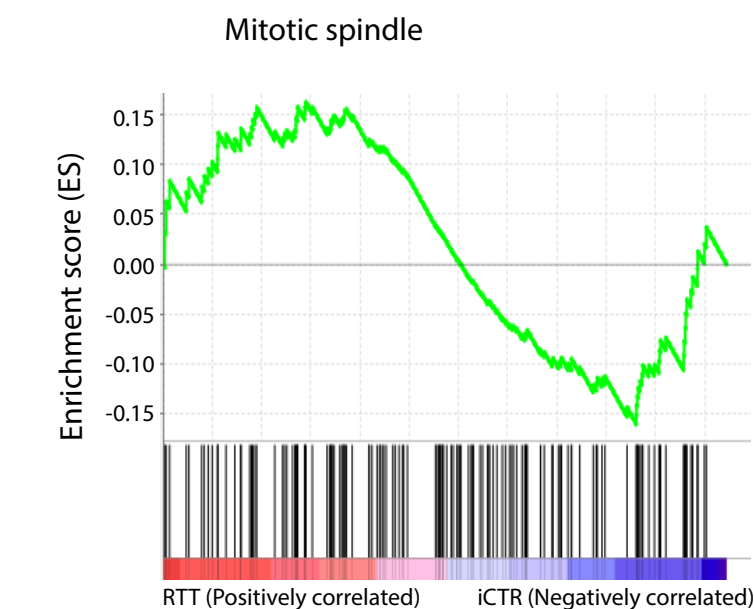

Supplement: Supplementary file 4 — Additional file 4: Figure S4. Top gene sets enriched in RTT-iPSCs. Proteins below p=0.1 are ranked by GSEA based on their differential expression level. Black vertical lines indicate the position where members of a pathway appear in the ranked gene list. [file 13229_2020_344_MOESM4_ESM.pdf]
