# Supplementary material for: Quantitative proteomic analysis of Rett iPSC-derived neuronal progenitors
Source: Mol Autism. 2020 May 27;11:38. doi: 10.1186/s13229-020-00344-3 (PMC7251722; doi:10.1186/s13229-020-00344-3)
Supplement: Supplementary file 3 — Additional file 3: Figure S3. Venn diagram. a. Number of proteins decreased expressed in RTT at different time points. b. Number of proteins increased expressed in RTT at different time points. c. Overview of the number of proteins altered in RTT. [file 13229_2020_344_MOESM3_ESM.pdf]

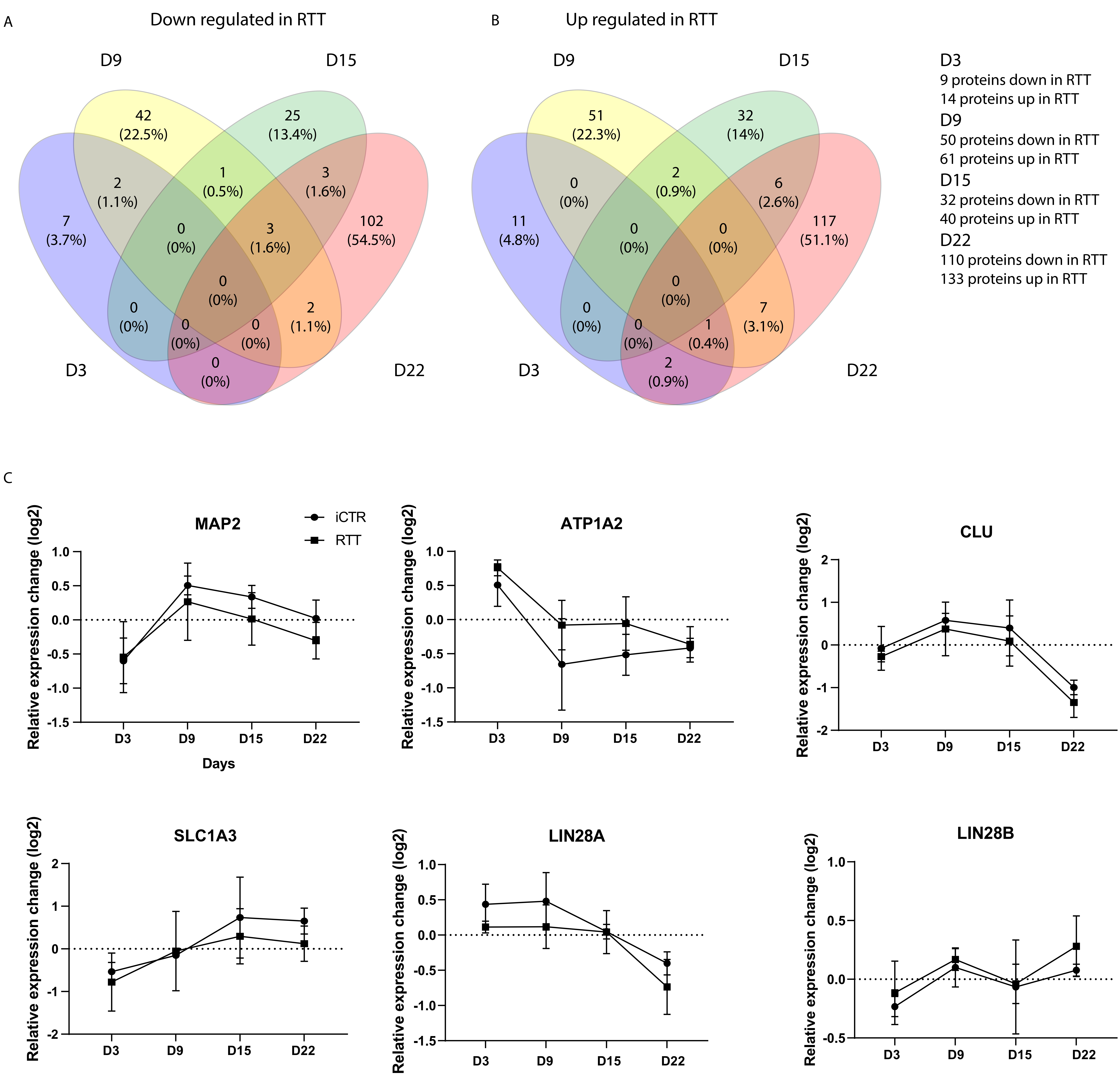

A
Down regulated in RTT
D9
D15
D3
D22
42
(22.5%)
25
(13.4%)
2
(1.1%)
1
(0.5%)
3
(1.6%)
7
(3.7%)
0
(0%)
3
(1.6%)
102
(54.5%)
0
(0%)
0
(0%)
0
(0%)
0
(0%)
2
(1.1%)
0
(0%)
B
Up regulated in RTT
D9
D15
D3
D22
51
(22.3%)
32
(14%)
0
(0%)
2
(0.9%)
6
(2.6%)
11
(4.8%)
0
(0%)
0
(0%)
117
(51.1%)
0
(0%)
0
(0%)
0
(0%)
1
(0.4%)
7
(3.1%)
2
(0.9%)
D3
9 proteins down in RTT
14 proteins up in RTT
D9
50 proteins down in RTT
61 proteins up in RTT
D15
32 proteins down in RTT
40 proteins up in RTT
D22
110 proteins down in RTT
133 proteins up in RTT
C
MAP2
iCTR
RTT
Relative expression change (log2)
Days
ATP1A2
CLU
SLC1A3
LIN28A
LIN28B
D3
D9
D15
D22
